# The effect of dietary antioxidant supplementation in a vertebrate host on the infection dynamics and transmission of avian malaria to the vector

**DOI:** 10.1007/s00436-018-5869-8

**Published:** 2018-05-09

**Authors:** Jessica Delhaye, Olivier Glaizot, Philippe Christe

**Affiliations:** 1 Department of Ecology and Evolution, Faculty of Biology and Medicine, University of Lausanne, CH-1015 Lausanne, Switzerland; 2 Museum of Zoology, Palais de Rumine, CH-1014 Lausanne, Switzerland

**Keywords:** *Culex pipiens*, Haemosporidian, Oxidative status, *Plasmodium relictum*, *Serinus canaria*

## Abstract

Host susceptibility to parasites is likely to be influenced by intrinsic factors, such as host oxidative status determined by the balance between pro-oxidant production and antioxidant defences. As a result, host oxidative status acts as an environmental factor for parasites and may constrain parasite development. We evaluated the role of host oxidative status on infection dynamics of an avian malarial parasite by providing canaries (*Serinus canaria*) with an antioxidant supplementation composed of vitamin E (a lipophilic antioxidant) and olive oil, a source of monounsaturated fatty acids. Another group received a standard, non-supplemented food. Half of the birds in each group where then infected with the haemosporidian parasite, *Plasmodium relictum*. We monitored the parasitaemia, haematocrit level, and red cell membrane resistance, as well as the transmission success of the parasite to its mosquito vector, *Culex pipiens*. During the acute phase, the negative effect of the infection was more severe in the supplemented group, as shown by a lower haematocrit level. Parasitaemia was lower in the supplemented group during the chronic phase only. Mosquitoes fed on supplemented hosts were more often infected than mosquitoes fed on the control group. These results suggest that dietary antioxidant supplementation conferred protection against *Plasmodium* in the long term, at the expense of a short-term negative effect. Malaria parasites may take advantage of antioxidants, as shown by the increased transmission rate in the supplemented group. Overall, our results suggest an important role of oxidative status in infection outcome and parasite transmission.

**Electronic supplementary material:**

The online version of this article (10.1007/s00436-018-5869-8) contains supplementary material, which is available to authorized users.

## Introduction

Inter-individual variation in susceptibility to a parasite is likely to depend on variations in a host’s intrinsic factors (Bichet et al. 2014). For instance, infection intensity may be positively correlated to host nutritional status when parasites take advantage of host resources for their own development (Christe et al. 2003; Pulkkinen and Ebert 2004). Alternatively, a poor host nutritional status may allow a higher infection intensity (Cornet et al. 2014), by impairing the host’s immune functions (Chandra 1996; Christe et al. 1998). Parasites may also adapt to the environment provided by their hosts. For example, *Plasmodium* parasites were shown to better exploit subsequent hosts when they first grew on control hosts than parasites grown on diet-enriched ones (Cornet et al. 2014). Host oxidative status may influence parasite development as well. The oxidative status of an organism is determined by the levels of pro-oxidant production and antioxidant defences. Physiological processes release and use pro-oxidants, which are involved in a variety of pathways from cell signalling (Apel and Hirt 2004; Finkel 2011) to parasite resistance (Nathan and Cunningham-Bussel 2013; Wink et al. 2011). Nonetheless, these pro-oxidants may also cause oxidative damage to lipids, proteins, and DNA and harm the host (Halliwell and Gutteridge 2007). Antioxidant defences (molecular and structural) protect the host by limiting oxidation or by repairing damage (reviewed in Pamplona and Costantini 2011). Endoparasites such as malaria parasites develop inside their host and are thus exposed to this pro-oxidant/antioxidant environment. As a result, host oxidative status is expected to act as an environmental factor that may constrain parasite development.

The relationship between a host’s oxidative status and its ability to fight against parasites is complex. Host immune defences partly rely on pro-oxidant compounds to which parasites are susceptible (Delhaye et al. 2016a). For example, it has been shown that high pro-oxidant levels impaired malaria parasite development (reviewed in Postma et al. 1996) and that inhibition of nitric oxide synthase, an enzyme responsible for the production of a pro-oxidant involved in resistance to parasites (Wink et al. 2011), increased host susceptibility to *Plasmodium* (Bichet et al. 2012). In contrast, high pro-oxidant levels have also been correlated with a decreased immune activation (Tobler et al. 2011), suggesting a higher susceptibility to parasites. Immune response may induce oxidative damage to the host (Bertrand et al. 2006) and may thus increase host susceptibility to the parasite (Delhaye et al. 2018). On the other hand, antioxidants may protect the host from collateral damage during the immune response (Costantini and Møller 2009). However, it has also been shown that a high superoxide dismutase level, an enzyme responsible of the detoxification of the superoxide pro-oxidant, was associated with a lower immune response (Cram et al. 2015), possibly through the anti-inflammatory property of this particular antioxidant defence. Finally, host antioxidants may also benefit the parasite by directly protecting it from host oxidative attack (Postma et al. 1996). Therefore, host oxidative status may influence both parasite development and host susceptibility.

In this study, we investigated the role of host antioxidant defences on parasite infection dynamics and transmission success in an avian malaria system. In order to examine this question, we used the canary, *Serinus canaria*, the haemosporidian parasite *Plasmodium relictum* (lineage SGS1), and its natural vector in the field *Culex pipiens* (Glaizot et al. 2012; Lalubin et al. 2013, 2014). We performed experimental manipulations of antioxidant defence properties of bird red blood cells by a dietary antioxidant supplementation. We provided olive oil, a source of monounsatured fatty acids that can be selectively (Buttemer et al. 2008) incorporated to biomembranes (Glatz et al. 1989), making them more resistant to oxidative damages (Bielski et al. 1983; Hulbert et al. 2007; Pamplona et al. 2002) and vitamin E, a lipophilic antioxidant protecting lipids (Tappel 1962) and consequently biomembranes (reviewed in Elsayed 2001). On the one hand, supplementation was expected to increase antioxidant defences and therefore to reduce the cost of infection and on the other hand to protect the parasite from host oxidative attack. We thus predicted a higher parasite intensity (i.e. parasitaemia), but a higher haematocrit level in supplemented hosts compared to control ones. Finally, because parasitaemia positively correlates to the probability of being transmitted to the mosquitoes (Vezilier et al. 2010), we predicted a higher transmission success in mosquitoes fed on supplemented infected birds.

## Methods

### Biological system

*P. relictum* (lineage SGS1) is the aetiological agent of the most prevalent form of avian malaria, commonly found infecting Passeriform birds in Europe (Valkiunas 2005). To our knowledge, it is also the only experimental (non-human) malaria model that brings together a mosquito-*Plasmodium* combination with a common evolutionary history. Our parasite lineage (SGS1) was isolated from infected great tits caught in the region of Lausanne (Switzerland) and transferred to canaries who have never been exposed to avian malaria (immunologically naïve) in the laboratory.

### Host antioxidant and infection treatments

Thirty-four 1-year old canaries (16 females and 18 males) were kept per group of four to five individuals of the same sex in eight cages (1 × 1 × 2 m) in the laboratory (14:10 day-night light cycle, 20 °C, 55% humidity). All individuals were uninfected prior the experiments as tested with two blood samples (1 month before and on the first day of the experiment). Birds were prepared for the experiment during 1 month as follows: each cage received fresh food (mix of 8 g of seeds Vitabalance and 6 g of couscous Migros Bio per individual) and fresh water (500 mL per cage) daily, distributed in two feeders and two water bowls. Once a week, the water was enriched with a vitamin mix covering the birds’ needs in vitamin (Océvit, Virbac, 1 mL per litre of water, according to the manufacturer’s recommendations). Half of the cages (balanced per sex) received a daily supplementation of dietary antioxidants: vitamin E (Océférol, Virbac, 1 mL per litre of water) and olive oil (Italian olive oil Migros Bio, 66 μL per gram of couscous). We calculated the supplementation based on the natural vitamin E contents of the standard canary food and on indications provided by the manufacturers. We added olive oil to the mix of seeds and couscous to increase the vitamin E content of the mix by about 50% (vitamin E content in seeds: 0.02 mg/g; in couscous: 1 μg/g; in olive oil: 0.12 mg/mL; information provided by the manufacturers). Concerning the supplementation in the water, we adapted the manufacturer’s recommendations for birds during the breeding season (vitamin E content in Océférol solution: 0.04 g/mL).

After 1 month, half of the birds (balanced per sex and per antioxidant treatment) were experimentally infected with *P. relictum* via an intraperitoneal injection of 75 μL of a pool of infected blood mixed with PBS (1:1). Parasitaemia was measured by quantitative PCR, which provides relative infection intensities. The absolute number of parasites injected in each canary was unknown. However, each canary received a given fraction of the same infected blood mix, which insured homogeneous inoculations among individuals. The other half of the birds received an injection of PBS only (control group). Following the experimental infection, each group of birds in a cage was divided into two groups of two to three canaries (16 cages) such as to reduce the number of individuals per cage in order to measure individual daily food consumption. After experimental infection and until the end of the experiment, antioxidant treatment was maintained and daily food consumption was measured by weighing the food remaining in the feeders each morning (9:00 ± 1:00). The food fallen to the ground among feathers, sand, and spilled water was counted as consumed food. The amount was expected to be proportional to the number of birds per cage. Individual daily food consumption was then estimated by dividing the daily food consumption per cage by the number of canaries in the cage; this final number was included in the relevant statistical analyses. The experiment was designed in two temporal blocks spaced a week with equivalent representation of sex, antioxidant, and infection treatments per block.

In order to determine the effect of the dietary antioxidant supplementation on bird red blood cell membrane resistance to oxidative attacks, birds were weighed and blood sampled prior to and 1 month after the beginning of the antioxidant treatment. The infection dynamics and the progression of cell membrane resistance were followed by sampling birds at 5, 12, 22, 33, and 42 days post-infection (dpi). Immediately after each sampling, 8 μL of blood was transferred in 292 μL of KRL buffer (Kirial international, Laboratoires Spiral S.A., Dijon, France; Alonso-Alvarez et al. 2004), a physiological buffer adjusted to bird cell osmolarity, and kept in the dark at 4 °C until laboratory measurement of cell membrane resistance (performed 4 h maximum after blood sampling). Haematocrit was measured in capillaries after 10 min of centrifugation at 12800 rpm and was expressed as the fraction of red blood cells in the total blood volume after processing. The rest of the blood was centrifuged for 10 min at 4 °C and 15,000 rcf. Red blood cells were stored at − 20 °C for *Plasmodium* detection and quantification.

### Parasite transmission to the vector

We collected *C. pipiens* egg rafts from plastic tanks (50 × 30 × 25 cm) filled up with water from Lake Geneva and baited with live yeast set up in the forest of Dorigny (46° 31′ N; 6° 34′ E; alt. 380 m). In the laboratory (24 °C, 65% relative humidity, and a 14:10-h light-dark cycle), clutches were allowed to hatch in individual containers filled up with 250 mL of mineral water and larvae were fed with commercial fish flakes (Tetra). Fourteen days before feeding on birds, daily emerging females were pooled together for 4 days in common cages (30 × 30 × 90 cm) and provided with a 10% glucose solution (four groups). Each canary was exposed to mosquitoes during the peak of parasitaemia during the acute phase of *Plasmodium* infection (14 ± 1 days post-infection; Cellier-Holzem et al. 2010), and each female mosquito took a blood meal at the same age (12.5 ± 1.5 days). Twenty-four hours before feeding, female mosquitoes were isolated per groups of 20 in cages (30 × 30 × 30 cm) and provided with water only. Each canary was presented to a group of 20 mosquitoes for 30 min three successive times. Engorged females (mean number of engorged females per bird ± standard deviation: infected control group: 10.9 ± 4.0; infected supplemented group: 12.7 ± 5.7) were then isolated in tubes (Sartsdet, 30 mL) and provided with a 10% glucose solution for 4 days in order to collect haematin, the excretion used to assess blood meal size. On day 5 post-feeding, females were transferred to a new tube with 2.5 mL of mineral water for oviposition and fed with a 10% glucose solution. Mosquitoes in the tubes were checked daily for survival. If still alive 26 days post-feeding, females were sacrificed. Naturally dead and sacrificed females were stored at − 80 °C until laboratory processing. Haematin collection tubes were stored at − 80 °C until quantification (as described in Briegel 1980; Rivero and Ferguson 2003). Wings were measured post-mortem (as described in Mpho et al. 2000) and used as a proxy for mosquito body size.

### Red blood cell membrane resistance to oxidative attack

We measured red blood cell membrane resistance to oxidative attack as the time (in minutes) needed to haemolyse half of the red blood cells in presence of a ROS-generating solution (as described in Bize et al. 2008). Cell haemolysis was quantified using a microplate reader following the decrease of optical density at the wavelength of 540 nm using the Kirial International processing analysis software.

### *Plasmodium* detection and quantification

Bird DNA was extracted from the red blood cells and mosquito DNA was extracted from the thorax using the DNeasy blood and tissue extraction kit (Qiagen, California), according to the manufacturer’s protocol. *Plasmodium* parasites were detected using PCR method previously developed by Waldenström et al. (2004). Parasite quantification in the vertebrate blood was performed using quantitative PCR (as described in Jenkins et al. 2015). Briefly, for both the parasite and the host, DNA concentration was calculated from a standard curve and the parasitaemia was given by the ratio of the parasite DNA concentration on the host DNA concentration. Parasitaemia, log transformed to achieve normality, increased from − 3.26 to 1.50 (unitless) and uninfected individuals had no value of parasitaemia.

### Statistical analyses

Statistical analyses were performed with R (version 3.1; R Development Core Team 2011). To estimate if there was a change in physiological and body condition parameters after the month of antioxidant treatment, we analysed red blood cell membrane resistance to oxidative attack, haematocrit, and body mass as response variables in linear mixed effect models (lme function in nlme package), including terms for time (prior to–1 month after the antioxidant treatment), antioxidant treatment (control-supplemented), sex (female-male), and the two-way interaction between time and antioxidant treatment. Subsequently, we analysed parasitaemia as a response variable in a linear mixed effect model including terms for time, antioxidant treatment, sex, all the two-way interactions, as well as the interaction between cubic time and antioxidant treatment. Following a first exposure to *Plasmodium*, the infection dynamics are characterised by a rapid increase of parasitaemia followed by a rapid decrease (acute phase of infection) ending with a stabilisation of parasitaemia at a lower level (chronic phase of infection, Cellier-Holzem et al. 2010). Adding cubic time to the model allows to account for these non-linear temporal dynamics. We compared the models fitting time only, squared time, or cubic time using AIC comparison of the full models (Galwey 2014), and the model with cubic time was retained (full model AIC fitting time only: 309.18; squared time: 272.16; cubic time: 227.39). We also tested the effect of experimental infection on the change of physiological and body condition parameters over time. We analysed red blood cell membrane resistance to oxidative attack, haematocrit, and body mass as response variables in linear mixed effect models including terms for time, antioxidant treatment, infection treatment (uninfected-infected), sex, as well as all the two-way interactions. As we were interested in the effect of the antioxidant treatment on the infection dynamics, we also added the interaction term between cubic time, antioxidant treatment, and infection treatment. To account for the repeated measurements, we implemented each model with an autocorrelation structure and added canary identity nested in cage identity nested in block as a random factor. We analysed individual daily food consumption, measured at the cage level, as a response variable in a linear mixed effect model including terms for the number of canaries per cage, time, antioxidant treatment, infection treatment, sex, all the two-way interactions, as well as the interaction between square time, antioxidant treatment, and infection treatment. We added an autocorrelation structure and cage identity nested in block as a random factor. Finally, we analysed the infection probability of female mosquitoes fed on infected canaries as a binomial response variable in a generalised linear mixed effect model (glmer function in lme4 package) including terms for body size, blood meal size, bird parasitaemia, bird antioxidant treatment, as well as emergent group as a random factor. For each response variable, we determined the explanatory power of each fitted parameter by performing likelihood ratio tests following a standard backward selection procedure by sequential elimination of each fitted terms of similar order from the full model (Crawley 2007). Only significant terms were kept to reach the minimal adequate model. The significant *p* values given in the text come from the minimal adequate models and the non-significant *p* values come from the likelihood ratio tests prior to the elimination of the non-significant term from the model. To look at the effect of each significant term individually, contrast analyses were performed (Crawley 2007). All the details about each model structure are given in Tables 1 and 2 and Supplementary tables (from Supplementary Tables 1 to 5).

**Table 1:** Parasitaemia (log transformed, arbitrary unit) in infected birds. The number of individuals (*n*), the repeated measurements (days post-infection), and the model type that were used are indicated. Minimal adequate model is given in bold with intercept as well as estimates, standard errors (se), *t* values, and *p* values for each specific significant terms. Non-significant terms tested are given with the *p* value of the likelihood ratio test before being dropped-out of the model

Parasitaemia (log transformed)				
*n* = 17, days post-infection: 5, 12, 22, 33, 42				
Linear mixed effect model				
Predictors	Estimate	se	*t* value	*p* value
Intercept	2.5308	0.5028	5.03	< 0.001
Time	− 0.1137	0.0217	− 5.25	< 0.001
Antioxidant	0.2640	0.1026	2.57	0.021
Sex				0.793
Time:antioxidant	− 0.0177	0.0072	− 2.45	0.017
Time:sex				0.533
Antioxidant:sex				0.092
(Time)2	− 0.0069	0.0003	− 24.30	< 0.001
(Time)2:antioxidant				0.233
(Time)3	0.0005	0.0001	8.93	< 0.001
(Time)3:antioxidant				0.766

**Table 2:** Infection probability in fed female mosquitoes. The number of individuals (*n*) and the model type that were used are indicated. Minimal adequate model is given in bold with intercept as well as estimates, standard errors (se), *z* values, and *p* values for each specific significant terms. Non-significant terms tested are given with the *p* value of the likelihood ratio test before being dropped-out of the model

Infection probability				
*n* = 192				
Generalised linear mixed effect model				
	Estimate	se	*z* value	*p* value
Intercept	− 3.0165	0.7547	− 4.00	< 0.001
Bird antioxidant treatment	1.1488	0.5473	2.10	0.036
Body size				0.057
Blood meal size				0.067
Bird parasitaemia				0.216

## Results

### Antioxidant treatment prior to experimental infection

**Fig. 1:**
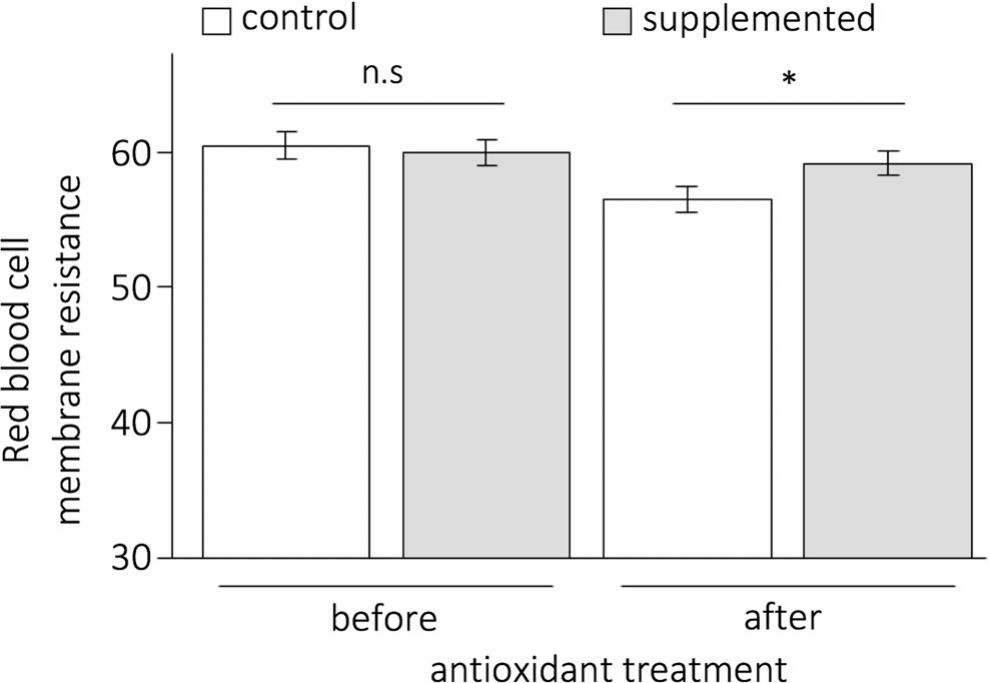
Red blood cell membrane resistance to oxidative attack (mean ± standard error, in minutes) prior to and 1 month after the antioxidant treatment for control and supplemented groups. Non-significant (n.s.) and significant (*) differences are indicated

Membrane resistance decreased in the control group but remained stable in the supplemented group resulting in a lower membrane resistance in the control group compared to the supplemented group prior to the experimental infection (*p* = 0.023, Fig. 1, Supplementary Table 1). Haematocrit and body mass were not affected by antioxidant treatment (*p* > 0.100), time (*p* > 0.100), or their interaction (*p* = 0.825). Sex had no effect per se on any of these variables (*p* > 0.200). Details of the models are summarised in Supplementary Table 1.

### Infection dynamics

On the 19 experimentally infected birds, two did not develop the infection, one control male and one supplemented female, and were thereby discarded from the analyses. At 5 days post-infection, five out of the remaining 17 experimentally infected birds, parasite was found to be infected with *P. relictum*: two supplemented females, two supplemented males, and one control male. The other 12 infected birds (i.e. two supplemented females, four control females, three supplemented males, and three control males), had too low parasitaemia at 5 dpi to be detected by qPCR. All experimentally infected birds had detectable parasitaemia at 12 dpi and at the subsequent sampling days. In order to run the statistical models on parasitaemia, we assumed that experimentally infected birds with undetectable parasitaemia at 5 dpi were parasitized at a low level. For this sampling day, they were thus given the smallest parasitaemia value detected by qPCR during the course of this experiment. Careful conclusions were drawn regarding the parasitaemia at that sampling point.

**Fig. 2:**
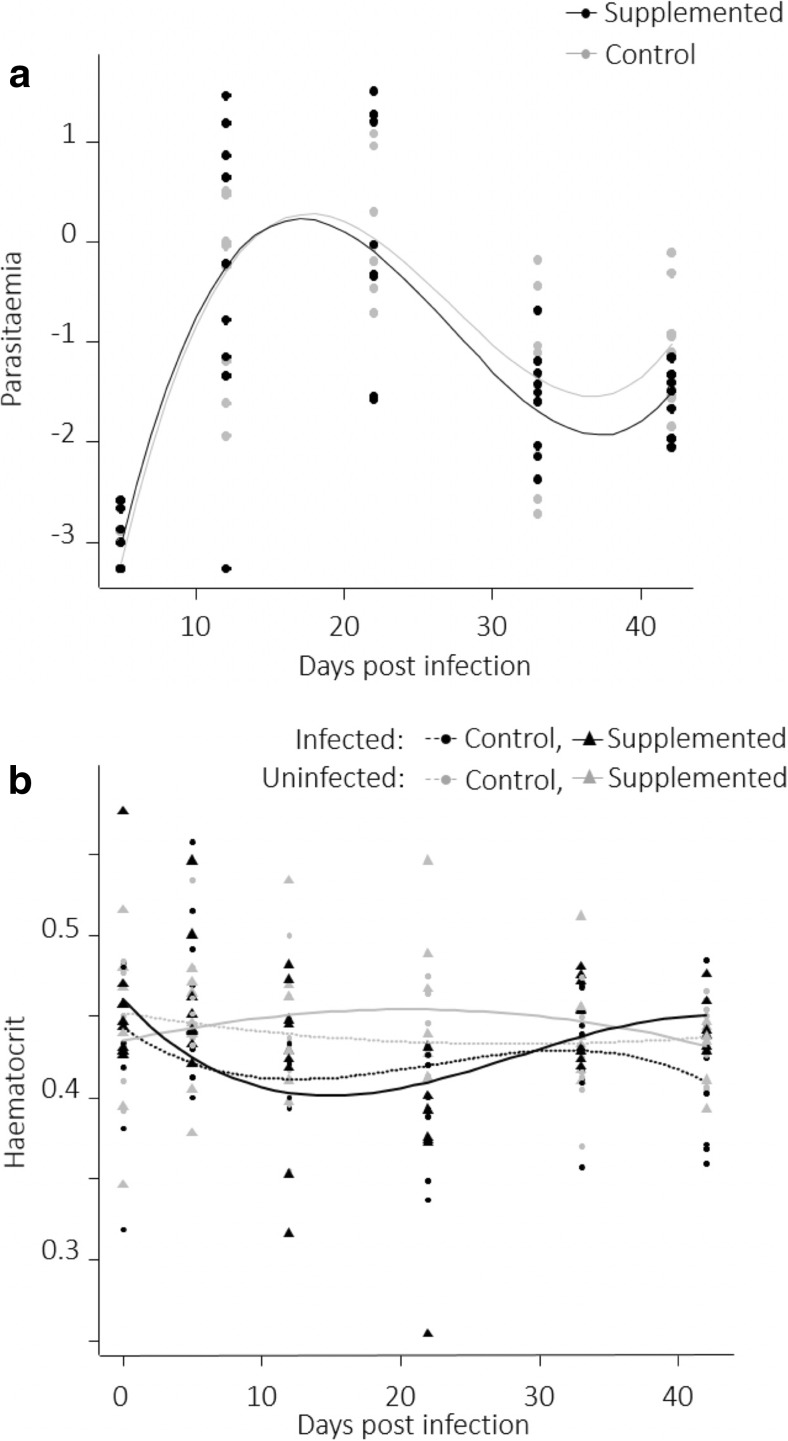
**a** Parasitaemia (log transformed, arbitrary units) in control and in supplemented infected birds as a function of days post infection. **b** Haematocrit (percent, %) as a function of antioxidant and infection treatments and days post infection

Parasitaemia increased until reaching a peak between 12 and 22 dpi (acute phase of infection) and decreased until reaching lower parasitaemia at 33 dpi remaining stable at 42 dpi (chronic phase of infection, *p* < 0.001, Fig. 2a, Table 1). In the chronic phase, supplemented birds had lower parasitaemia compared to control ones (*p* = 0.017, Fig. 2a, Table 1). The infection dynamics were accompanied by changes of bird haematocrit. Haematocrit of uninfected birds did not change significantly over time (Fig. 2b, Supplementary Table 2). However, the haematocrit of infected birds decreased as parasitaemia increased up until the peak of the acute phase and then increased with decreasing parasitaemia in the chronic phase (*p* = 0.005, Fig. 2b, Supplementary Table 2). At 42 dpi, supplemented infected birds had higher haematocrit compared to control infected ones (*p* = 0.006, Fig. 2b, Supplementary Table 2).

### Red blood cell membrane resistance to oxidative attack

**Fig. 3:**
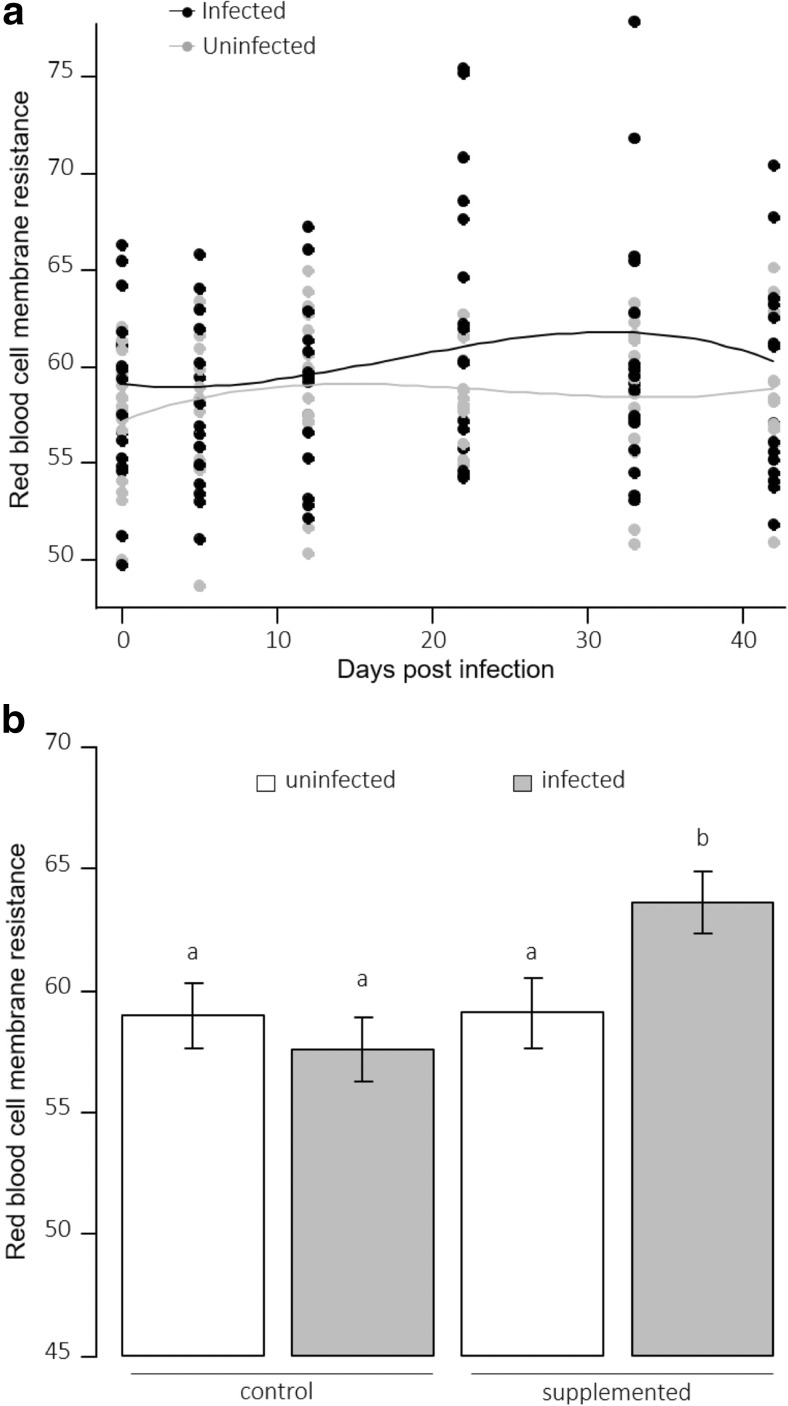
Red blood cell membrane resistance to oxidative attack (minutes) as a function of **a** days post-infection for uninfected and infected birds and **b** antioxidant and infection treatments (different letters indicate significant differences)

Membrane resistance of uninfected birds was stable over time and there was no difference between control and supplemented birds (Fig. 3a, Supplementary Table 3). Membrane resistance of infected birds increased after 12 dpi until reaching a peak between 22 and 33 dpi and decreased between 33 and 42 dpi (*p* = 0.005, Fig. 3a, Supplementary Table 3). It was higher in supplemented infected birds in comparison to the other groups (*p* = 0.023, Fig. 3b, Supplementary Table 3).

### Parasite transmission to the vector

**Fig. 4:**
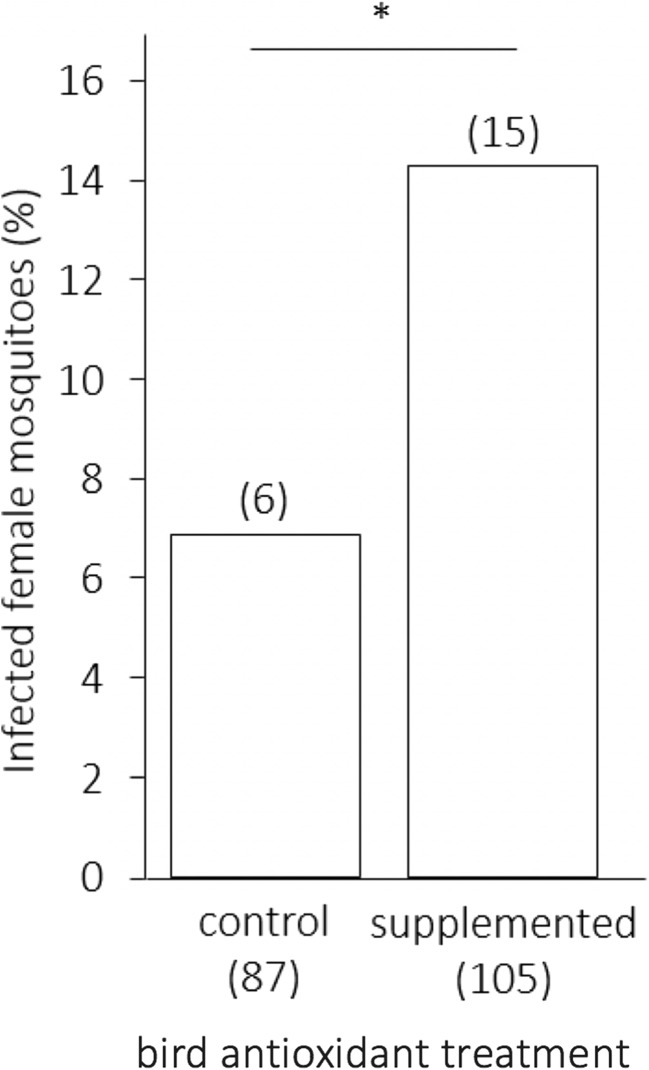
Prevalence of infected female mosquitoes (percent, %) as a function of the antioxidant treatment of the infected bird host. Sample sizes are given in brackets below each bar: the number of fed females and above each bar: the number of infected females. A star indicates a significant difference

Prevalence was almost twice higher in female mosquitoes fed on supplemented birds than in those fed on control ones (*p* = 0.036, Fig. 4, Table 2). There was no significant effect of bird parasitaemia, blood meal size, or mosquito body size on infection probability (Table 2).

### Food consumption

Individual daily food consumption varied with antioxidant and infection treatment (Supplementary Table 4). Birds from the control group ate more than birds from the supplemented group (mean in gram ± standard error: control: 8.19 ± 0.09, supplemented: 7.80 ± 0.09, *p* = 0.008, Supplementary Table 4), and uninfected birds ate more than infected ones (mean in gram ± standard error: uninfected: 8.17 ± 0.09, infected: 7.82 ± 0.09, *p* = 0.017, Supplementary Table 4). Food consumption decreased with number of individuals per cage (mean in gram ± standard error: two birds per cage: 8.31 ± 0.07, three birds per cage: 6.79 ± 0.12, *p* < 0.001, Supplementary Table 4). Body mass was neither affected by antioxidant (*p* = 0.204) nor by infection treatment (*p* = 0.878, Supplementary Table 5). Body mass followed a slight fluctuation with cubic time (*p* < 0.001, Supplementary Table 5).

## Discussion

### Infection dynamics and oxidative status in the vertebrate host

Diet richness has been shown to influence the infection dynamics of *Plasmodium*, more precisely to influence the peak of parasitaemia during the acute phase of infection with a higher parasitaemia in birds with a poorer diet (Cornet et al. 2014). Our results however show a different pattern since antioxidant treatment had an effect on parasitaemia only during the early and late phases of infection. After a month of the experiment, antioxidant supplementation resulted in a higher membrane resistance in supplemented birds compared to control ones. It was expected to help supplemented birds better cope with oxidative damage occurring during the struggle with *Plasmodium* but also to protect the parasite, thereby allowing it to develop a higher parasitaemia. At early infection (5 dpi), four out of the nine supplemented individuals had a parasitaemia high enough to be detected in the blood, compared to one out of the eight in the control group. This result suggests that oxidative status prior to infection may mediate early parasite development and that antioxidants may favour *P. relictum* development. During the acute phase of infection (at 12 dpi), supplemented birds suffered a haematocrit decrease despite similar parasitaemia compared to control birds, which suggests stronger pathogenic effects in supplemented birds. It has already been proposed that parasite development and pathogenic effects can be decoupled, as pathogenic effects may result from both parasite multiplication and the activation of the host’s immune system (Sorci 2013). For example, mounting an immune response after injection of an immune stimulator has been linked with oxidative damage to red blood cells (Bertrand et al. 2006). Supplemented birds may have had a higher immune response to control ones, resulting in a higher lysis of red blood cells and a lower haematocrit. This mechanism, called immunopathology (Graham et al. 2005), may lead to the selection of less virulent parasites because a high damage to the host can compromise the parasite’s transmission success (Sorci 2013). In the current experiment, infected birds received as inoculum of a pool of blood from several infected individuals which may have allowed inter-host heterogeneity in *Plasmodium* clone selection. This phenomenon could also explain the lower parasitaemia observed in the chronic phase of infection in supplemented birds in which selection against virulent parasites may have occurred. The potential effect of supplementation on host immune response can be explained by the role of fatty acids acting as fuel for lymphocytes and may thus be important for immune functions (reviewed in Pond 1996). Therefore, our results illustrate that the host oxidative status affects infection dynamics and pathogenic effects.

The present results were obtained following experimental infections that originated from intraperitoneal injections of asexual stages of *P. relictum*. Even though this practice is very common in laboratory infection experiments of *Plasmodium*, it nevertheless represents an artificial transmission route for the parasite. It is important to note that the infection dynamics, as well as the triggered host responses might exhibit different timing and/or amplitude with natural or experimental sporozoite infections.

Following the month of food supplementation, we observed a higher membrane resistance in supplemented birds compared to control ones. Surprisingly, this was not due to an increase in the supplemented group as expected, but due to a decrease in the control group. Although birds were maintained in the laboratory under controlled conditions, it is possible that they experienced an unintended physiological stress that the supplementation countered. Following experimental infections, membrane resistance of infected birds increased after the peak of parasitaemia, coinciding with an increase in haematocrit. This suggests either that a new pool of red blood cells was released in the circulation to restore normal haematocrit or that a turnover of lipids composing the cell membranes occurred (Giron-Calle et al. 1997). This was followed by a decreased resistance in the chronic phase that could be attributed to normal cell ageing and loss of membrane resistance. Overall, the supplemented infected birds had higher membrane resistance than the rest of the individuals and supplementation did not affect membrane resistance in the uninfected birds. This result supports the hypothesis that antioxidant supplementation may be advantageous only under oxidative stress conditions (Beaulieu and Schaefer 2013), such as a pathogenic infection, and highlights the importance of dietary antioxidants for cell membrane resistance during an infection with *Plasmodium*.

### Transmission success to the mosquito vector

Vertebrates can be dead end hosts for *Plasmodium* parasites if the parasites are not transmitted to a vector in which they undergo their sexual reproduction (Valkiunas 2005). The parasite intensity in the blood has been shown to affect life history traits of vectors (Delhaye et al. 2016b) and has been associated to the transmission probability to the vector, measured as oocyst prevalence and oocyst burden (Vezilier et al. 2010; Pigeault et al. 2015). It has also been shown that oocyst prevalence and oocyst burden depend on mosquito blood meal size (Pigeault et al. 2015). Here, we observed a higher parasite transmission success, measured as the presence of infectious sporozoite stages in the salivary glands, when mosquitoes fed on supplemented birds, without significant effect of mosquito blood meal size or bird parasitaemia. It has already been shown that vertebrate plasma components ingested during mosquito blood meal may affect parasite development in the vector (Gouagna et al. 2004; Lopes et al. 2007). Mosquito immune defences partly rely on pro-oxidant attack (Goncalves et al. 2012; Luckhart et al. 1998). During mosquito feeding, parasites may have been ingested with antioxidants present in the blood. It has also been shown that the human parasite *Plasmodium falciparum* integrates fatty acids from the vertebrate host resources (Mi-ichi et al. 2006), and that the type of lipids does not influence parasite growth (Frankland et al. 2007). Both processes may have conferred protection to the parasites in the mosquito. It is also possible that the supplementation favoured gametocyte production and enhanced parasite transmission to the vector. These three different processes may explain why despite similar parasitaemia in the acute phase under both antioxidant treatments, parasites are better transmitted to the vector when they developed in supplemented vertebrate hosts than in control ones. In any cases, this result indicates that vertebrate host oxidative status influences parasite transmission to the vector.

### Food consumption

Infected birds ate less but had similar body mass than uninfected ones independently of their diet. This may be explained by a higher lipid content in supplemented food and thus a higher energy efficiency. Decreased food consumption is a phenomenon that has been observed during several host-parasite associations (Crompton 1984). In addition, it has been shown that *P. falciparum* needs and takes a variety of host resources for its own (reviewed in LeRoux et al. 2009). Decreasing food intake may then help to fight against parasites through the reduction of host resources that parasites can divert for their own. The absence of an effect on body mass suggests that infected individuals were also less active and had therefore lower energetic needs. Anorexia and lethargy have been described as part of the vertebrate sickness behaviours that are known to be adaptive behavioural responses against parasites (Adelman and Martin 2009; Hart 1988). Thus, sickness behaviours may be part of host defence mechanisms during an infection by *Plasmodium*.

## Conclusion

We found that bird oxidative status influences the infection dynamics of *Plasmodium* in the vertebrate host as well as the parasite transmission to the vector. These results suggest that antioxidant availability may be one factor determining not only the strength of immune activation but also the magnitude of collateral damage. They also support the idea that parasite development is likely to be determined by host intrinsic factors, as suggested by Bichet et al. (2014). Furthermore, host environment may influence a host’s susceptibility to parasites as well as parasite transmission, by affecting host oxidative status (through food quality, pollutants, or other oxidative stress generators). This further supports to the importance of local environmental factors in epidemiology and disease transmission.

## Electronic supplementary material

ESM 1 (DOCX 54.5 kb)
