# Supplementary material for: The effect of dietary antioxidant supplementation in a vertebrate host on the infection dynamics and transmission of avian malaria to the vector
Source: Parasitol Res. 2018 May 9;117(7):2043–52. doi: 10.1007/s00436-018-5869-8 (PMC6006207; doi:10.1007/s00436-018-5869-8)

**The effect of dietary antioxidant supplementation in a vertebrate host on the infection dynamic and transmission of avian malaria to the vector**

Parasitology Research

Jessica Delhaye^1^; Olivier Glaizot^2*^ & Philippe Christe^1^

^1^Department of Ecology and Evolution, University of Lausanne, CH-1015 Lausanne, Switzerland

^2^Museum of zoology, Palais de Rumine, CH-1014 Lausanne, Switzerland

jessica.delhaye@live.fr (orcid.org/0000-0002-9849-1447)

olivier.glaizot@unil.ch; +4121 316 34 67; (orcid.org/0000-0001-9116-3355)

philippe.christe@unil.ch (orcid.org/0000-0002-8605-7002)

* Corresponding author

Supplementary Table 1. Physiological and body condition parameters prior to and one month after the dietary antioxidant supplementation: red blood cell membrane resistance to oxidative attack (minutes), haematocrit (fraction of red blood cells in the total blood volume) and body mass (grams). The number of individuals (n_individual_), the repeated measurements and the model type that were used are indicated. Minimal adequate models are given in bold with intercept as well as estimates, standard errors (se), t-values and p-values for each specific significant terms. Non-significant terms tested are given with the p-value of the likelihood ratio test before being dropped-out of the model.

| n_individual_: 34, prior to and after supplementation | | | | | | |
| --- | --- | --- | --- | --- | --- | --- |
| Linear mixed effect model | | |  | | | |
| **Red blood cell membrane resistance** | | Estimate | | se | t-value | p-value |
|  | **Intercept** | **60.4373** | | **0.9957** | **60.70** | **<0.001** |
|  | **Time** | **-3.9603** | | **0.9239** | **-4.29** | **<0.001** |
|  | **Antioxidant** | **-0.5071** | | **1.4026** | **-0.36** | **0.720** |
|  | Sex |  | |  |  | 0.803 |
|  | **Time:antioxidant** | **3.2014** | | **1.3375** | **2.39** | **0.023** |
| **Haematocrit** | |  | |  |  |  |
|  | Time |  | |  |  | 0.411 |
|  | Antioxidant |  | |  |  | 0.120 |
|  | Sex |  | |  |  | 0.835 |
|  | Time:antioxidant |  | |  |  | 0.950 |
| **Body mass** | |  | |  |  |  |
|  | Time |  | |  |  | 0.150 |
|  | Antioxidant |  | |  |  | 0.342 |
|  | Sex |  | |  |  | 0.246 |
|  | Time:antioxidant |  | |  |  | 0.194 |

Supplementary Table 2. Haematocrit (fraction of red blood cells in the total blood volume). The number of individuals (n_individual_), the repeated measurements (days post infection) and the model type that were used are indicated. Minimal adequate model is given in bold with intercept as well as estimates, standard errors (se), t-values and p-values for each specific significant terms. Non-significant terms tested are given with the p-value of the likelihood ratio test before being dropped-out of the model.

| **Haematocrit** | | | | | | |
| --- | --- | --- | --- | --- | --- | --- |
| n_individual_ = 32, days post infection: 0, 5, 12, 22, 33, 42 | | | | | | |
| Linear mixed effect model | | |  | | | |
|  | Predictors | Estimate | | se | t-value | p-value |
|  | **Intercept** | 0.4430 | | 0.0146 | 30.44 | <0.001 |
|  | **Time** | -0.0005 | | 0.0005 | -0.93 | 0.356 |
|  | **Antioxidant** | 0.0072 | | 0.0189 | 0.38 | 0.707 |
|  | **Infection** | -0.0495 | | 0.0202 | -2.45 | 0.021 |
|  | Sex |  | |  |  | 0.094 |
|  | **Time:antioxidant** | 0.0007 | | 0.0005 | 1.37 | 0.173 |
|  | **Time:infection** | 0.0016 | | 0.0007 | 2.37 | 0.019 |
|  | Time:sex |  | |  |  | 0.111 |
|  | **Antioxidant:infection** | -0.0207 | | 0.0258 | -0.80 | 0.430 |
|  | Antioxidant:sex |  | |  |  | 0.262 |
|  | Infection:sex |  | |  |  | 0.154 |
|  | **(Time)2** | 0.00003 | | 0.00003 | 0.78 | 0.435 |
|  | **(Time)2: antioxidant** | -0.00007 | | 0.00004 | -1.72 | 0.088 |
|  | **(Time)2:infection** | 0.00003 | | 0.00005 | 0.60 | 0.552 |
|  | **(Time)3** | -0.0000001 | | 0.000001 | -0.08 | 0.936 |
|  | **Time:antioxidant:infection** | -0.0006 | | 0.0007 | -0.86 | 0.389 |
|  | (Time)3:antioxidant |  | |  |  | 0.193 |
|  | **(Time)3:infection** | **-0.0000047** | | **0.000002** | **-2.87** | **0.005** |
|  | **(Time)2:antioxidant:infection** | **0.00016** | | **0.00006** | **2.79** | **0.006** |
|  | (Time)3:antioxidant:infection |  | |  |  | 0.601 |

Supplementary Table 3. Red blood cell membrane resistance to oxidative attack (minutes). The number of individuals (n_individual_), the repeated measurements (days post infection) and the model type that were used are indicated. Minimal adequate model is given in bold with intercept as well as estimates, standard errors (se), t-values and p-values for each specific significant terms. Non-significant terms tested are given with the p-value of the likelihood ratio test before being dropped-out of the model.

| **Red blood cell membrane resistance to oxidative attack** | | | | | | |
| --- | --- | --- | --- | --- | --- | --- |
| n_individual_ = 32, days post infection: 0, 5, 12, 22, 33, 42 | | | | | | |
| Linear mixed effect model | | |  | | | |
|  | Predictors | Estimate | | se | t-value | p-value |
|  | **Intercept** | 59.7034 | | 1.6552 | 36.07 | <0.001 |
|  | **Time** | -0.0372 | | 0.0519 | -0.72 | 0.475 |
|  | **Antioxidant** | 0.0883 | | 1.7943 | 0.05 | 0.961 |
|  | **Infection** | -4.9808 | | 2.3100 | -2.16 | 0.040 |
|  | Sex |  | |  |  | 0.906 |
|  | Time:antioxidant |  | |  |  | 0.812 |
|  | **Time:infection** | 0.1890 | | 0.0714 | 2.65 | 0.009 |
|  | Time:sex |  | |  |  | 0.313 |
|  | **Antioxidant:infection** | **5.9459** | | **2.4706** | **2.41** | **0.023** |
|  | Antioxidant:sex |  | |  |  | 0.703 |
|  | Infection:sex |  | |  |  | 0.710 |
|  | **(Time)2** | -0.0033 | | 0.0014 | -2.43 | 0.016 |
|  | (Time)2:antioxidant |  | |  |  | 0.093 |
|  | **(Time)2:infection** | 0.0021 | | 0.0019 | 1.14 | 0.256 |
|  | **(Time)3** | 0.0002 | | 0.0001 | 1.71 | 0.089 |
|  | Time:antioxidant:infection |  | |  |  | 0.072 |
|  | (Time)3:antioxidant |  | |  |  | 0.400 |
|  | **(Time)3:infection** | **-0.0005** | | **0.0001** | **-2.87** | **0.005** |
|  | (Time)2:antioxidant:infection |  | |  |  | 0.391 |
|  | (Time)3:antioxidant:infection |  | |  |  | 0.773 |

Supplementary Table 4. Individual daily food consumption (in gram per individual per day). The number of cages (n_cage_), the repeated measurements (days post infection) and the model type that were used are indicated. Minimal adequate model is given in bold with intercept as well as estimates, standard errors (se), t-values and p-values for each specific significant terms. Non-significant terms tested are given with the p-value of the likelihood ratio test before being dropped-out of the model.

| **Individual daily food consumption** | | | | | | |
| --- | --- | --- | --- | --- | --- | --- |
| n_cage_ = 16, days post infection: from 0 to 42 | | | | | | |
| Linear mixed effect model | | |  | | | |
|  | Predictors | Estimate | | se | t-value | p-value |
|  | **Intercept** | 11.6356 | | 0.2816 | 41.32 | <0.001 |
|  | **Number of canary per cage** | **-1.5108** | | **0.1297** | **-11.65** | **<0.001** |
|  | **Time** | 0.0031 | | 0.0020 | 1.52 | 0.128 |
|  | **Antioxidant** | **-0.3935** | | **0.1248** | **-3.15** | **0.008** |
|  | **Infection** | **-0.3547** | | **0.1300** | **-2.73** | **0.017** |
|  | Sex |  | |  |  | 0.854 |
|  | Time:antioxidant |  | |  |  | 0.148 |
|  | Time:infection |  | |  |  | 0.367 |
|  | Time:sex |  | |  |  | 0.733 |
|  | Antioxidant:infection |  | |  |  | 0.980 |
|  | Antioxidant:sex |  | |  |  | 0.052 |
|  | Infection:sex |  | |  |  | 0.826 |
|  | **(Time)2** | **-0.0007** | | **0.0002** | **-3.81** | **<0.001** |
|  | (Time)2:antioxidant |  | |  |  | 0.840 |
|  | (Time)2:infection |  | |  |  | 0.628 |
|  | Time:antioxidant:infection |  | |  |  | 0.071 |
|  | (Time)2:antioxidant:infection |  | |  |  | 0.131 |

Supplementary Table 5. Body mass (in grams). The number of individuals (n_individual_), the repeated measurements (days post infection) and the model type that were used are indicated. Minimal adequate model is given in bold with intercept as well as estimates, standard errors (se), t-values and p-values for each specific significant terms. Non-significant terms tested are given with the p-value of the likelihood ratio test before being dropped-out of the model.

| **Body mass** | | | | | | |
| --- | --- | --- | --- | --- | --- | --- |
| n_individual_ = 32, days post infection: 0, 5, 12, 22, 33, 42 | | | | | | |
| Linear mixed effect model | | |  | | | |
|  | Predictors | Estimate | | se | t-value | p-value |
|  | **Intercept** | 23.8337 | | 0.5298 | 44.98 | <0.001 |
|  | **Time** | 0.0228 | | 0.0097 | 2.36 | 0.020 |
|  | Antioxidant |  | |  |  | 0.204 |
|  | Infection |  | |  |  | 0.878 |
|  | Sex |  | |  |  | 0.249 |
|  | Time:antioxidant |  | |  |  | 0.065 |
|  | Time:infection |  | |  |  | 0.593 |
|  | Time:sex |  | |  |  | 0.250 |
|  | Antioxidant:infection |  | |  |  | 0.693 |
|  | Antioxidant:sex |  | |  |  | 0.342 |
|  | Infection:sex |  | |  |  | 0.777 |
|  | **(Time)2** | 0.0017 | | 0.0003 | 5.72 | <0.001 |
|  | (Time)2:antioxidant |  | |  |  | 0.188 |
|  | (Time)2:infection |  | |  |  | 0.581 |
|  | **(Time)3** | **-0.00010** | | **0.00002** | **-4.25** | **<0.001** |
|  | Time:antioxidant:infection |  | |  |  | 0.079 |
|  | (Time)3:antioxidant |  | |  |  | 0.432 |
|  | (Time)3:infection |  | |  |  | 0.445 |
|  | (Time)2:antioxidant:infection |  | |  |  | 0.636 |
|  | (Time)3:antioxidant:infection |  | |  |  | 0.542 |

Supplementary Figure 1. Body mass (in gram) as a function of days post inoculation.


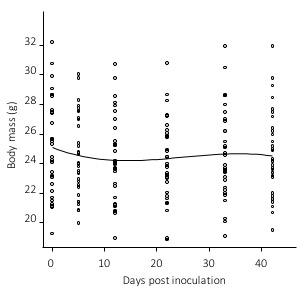

Supplement: Supplementary file 1 — (DOCX 54.5 kb) [file 436_2018_5869_MOESM1_ESM.docx]
